# A central storage facility to reduce pesticide suicides - a feasibility study from India

**DOI:** 10.1186/1471-2458-13-850

**Published:** 2013-09-16

**Authors:** Lakshmi Vijayakumar, Lakshmanan Jeyaseelan, Shuba Kumar, Rani Mohanraj, Shanmugasundaram Devika, Sarojini Manikandan

**Affiliations:** 1 Sneha, Voluntary Health Services, Chennai, India; 2 University of Melbourne, Parkville, Australia; 3 Griffith University, Brisbane, Australia; 4 Department of Bio-statistics, Christian Medical College, Vellore, India; 5 Samarth, 100, Warren Road, Chennai, India; 6 Raju Nursing Home, Usman Road, T. Nagar, Chennai, India

**Keywords:** Pesticides, Suicide, Centralized storage facility, Community intervention

## Abstract

**Background:**

Pesticide suicides are considered the single most important means of suicide worldwide. Centralized pesticide storage facilities have the possible advantage of delaying access to pesticides thereby reducing suicides. We undertook this study to examine the feasibility and acceptability of a centralized pesticide storage facility as a preventive intervention strategy in reducing pesticide suicides.

**Methods:**

A community randomized controlled feasibility study using a mixed methods approach involving a household survey; focus group discussions (FGDs) and surveillance were undertaken. The study was carried out in a district in southern India. Eight villages that engaged in floriculture were identified. Using the lottery method two were randomized to be the intervention sites and two villages constituted the control site. Two centralized storage facilities were constructed with local involvement and lockable storage boxes were constructed. The household survey conducted at baseline and one and a half years later documented information on sociodemographic data, pesticide usage, storage and suicides.

**Results:**

At baseline 4446 individuals (1097 households) in the intervention and 3307 individuals (782 households) in the control sites were recruited while at follow up there were 4308 individuals (1063 households) in the intervention and 2673 individuals (632 households) in the control sites. There were differences in baseline characteristics and imbalances in the prevalence of suicides between intervention and control sites as this was a small feasibility study.

The results from the FGDs revealed that most participants found the storage facility to be both useful and acceptable. In addition to protecting against wastage, they felt that it had also helped prevent pesticide suicides as the pesticides stored here were not as easily and readily accessible. The primary analyses were done on an Intention to Treat basis. Following the intervention, the differences between sites in changes in combined, completed and attempted suicide rates per 100,000 person-years were 295 (95% CI: 154.7, 434.8; p < 0.001) for pesticide suicide and 339 (95% CI: 165.3, 513.2, p < 0.001) for suicide of all methods.

**Conclusions:**

Suicide by pesticides poisoning is a major public health problem and needs innovative interventions to address it. This study, the first of its kind in the world, examined the feasibility of a central storage facility as a means of limiting access to pesticides and, has provided preliminary results on its usefulness. These results need to be interpreted with caution in view of the imbalances between sites. The facility was found to be acceptable, thereby underscoring the need for larger studies for a longer duration.

**Trial registration:**

ISRCTN: ISRCTN04912407

## Background

The W.H.O considers that the single most important means of suicide worldwide is by ingestion of pesticides and accounts for 1/3^rd^ of all suicides [1]. Gunnell et al. [2] surmised that at least 233,997 to 325,907 suicides per year are by pesticide poisoning. Mortality data on international suicide patterns revealed that in Asia, rural Latin American countries and Portugal, pesticide suicide was a major problem, notably among women [3].

Studies from Asia have found that pesticide suicides are impulsive acts, undertaken during stressful life events and majority of them do not suffer from mental disorders [4-6]. Case fatalities from pesticide poisoning are estimated to be between 10 – 20% in Asian countries [7]. In the above context, restricting access to pesticides can be an effective and relatively simple approach to prevent suicides. Gunnell et al. [8] have shown that restricting sales of highly toxic pesticides has coincided with a reduction in suicides in Sri Lanka. Hawton et al. [9] found that, the introduction of individual lockable boxes for storing pesticides in farming households in Sri Lanka was acceptable. Konradsen et al. [10,11] reported that lockable boxes were beneficial as it protected the pesticides from exposure to sun and rain and reduced the risk of theft. However, the authors went on to state that while the lockable storage boxes had enhanced safety, particularly for children, the introduction of these storage boxes had resulted in the farmers shifting from storing the pesticides in the fields to the home. This, the authors cautioned could increase the risk of impulsive self-poisoning as the pesticides were now more easily accessible. More recently, Patel et al. [12] in a nationally representative study from India reported that suicides in 49% men and 44% women were primarily through pesticide poisoning which is much higher than data provided by the government [13].

Therefore, the idea of a central location in the village where each family has its own locker to store their pesticides has the advantage of i) reducing storage of pesticides in homes and fields thereby restricting accessibility (ii) enabling involvement of the entire village or community (iii) permitting easy monitoring of the facility and (iv) enhancing cost effectiveness. There are also potential disadvantages of a central storage facility, namely that its location may not be accessible to all farmers in a particular village, the supervisors may not be present all the time making it inconvenient for farmers to use the facility and there is also the potential for misuse of the pesticides stored in the facility in the absence of proper supervisory checks. We undertook this study to examine the feasibility and acceptability of a centralized pesticide storage facility, as a possible preventive intervention strategy in reducing pesticide related suicides.

## Methods

### Study design and participants

A community randomized controlled feasibility study using a mixed methods approach involving focus group discussions (FGD), household survey and surveillance was undertaken. One consenting adult (over 18 years of age) who was either the head or the main earning member, or an adult son or wife of the head of the household constituted the key respondent to the survey. Non-resident individuals or those suffering from poor mental or physical health were not included as respondents. Ethical approval for the study was obtained from the Institutional Review Board of the Voluntary Health Services. The Voluntary Health Services is a 500 bedded multi specialty community hospital founded in Chennai over 50 years ago. It provides subsidized care to the socially weaker section of the society. The hospital has been involved in large scale research in HIV/AIDS, community mental, physical and maternal health.

### Study sites

The study was carried out in Kattumannarkoil Taluk, Cuddalore district, Tamilnadu state in Southern India which is about 5 hours from Chennai city. We visited the District Revenue Office (DRO) from where we obtained information on the villages governed by this taluk. We learnt that the taluk administers 161 villages. The number of households in each village ranges from a minimum of about 500 to around 1000. While the majority of the villages cultivate paddy, teak etc., a few of them are primarily engaged in floriculture (eg. Jasmine, Kanakambaram and Mullai etc.). Eight villages were identified as predominantly engaging in floriculture which requires spraying of pesticides twice a month resulting in higher and frequent pesticide usage. The lottery method was used to select four villages. The first two villages were allocated for intervention and the second two became the controls. As per the data provided by the DRO, the villages of Kandamangalam and Kurungudi (intervention villages) had 935 and 693 households respectively (Total: 1628 households) and the villages of Pazhanjanallur and Karunagaranallur with 835 and 541 households respectively (Total: 1376 households), constituted the control sites.

### Focus group discussions

Focus group discussions (FGDs) were carried out separately with men and women to understand their perceptions on suicidal behaviour and the concept of central storage of pesticides. Purposive sampling technique was used in recruiting adult (over 18 yrs) men and women who gave consent to participate. To be eligible for inclusion, participants had to be residents in the selected villages and engaged in floriculture thereby enabling them to talk about issues concerning pesticide use in the village. Permission to tape-record the discussions was sought before the conduct of the group. The FGDs were conducted at baseline i.e. before commencement of the intervention and again following completion of one year of the intervention.

A total of 8 FGDs in the intervention site and 8 in the control site, (4 each with men and women respectively), leading to a total of 16 FGDs altogether were carried out during the baseline survey. At follow - up the number of FGDs was halved with 4 conducted in the intervention sites and 4 in the control sites (2 each with men and women respectively). A Focus group guide was developed to ensure that all issues were consistently discussed.

All FGDs were conducted in Tamil by trained, gender specific social scientists assisted by a note taker. Care was taken to ensure that all the core elements in the guide were adequately and comprehensively addressed. New issues emerging during the discussions were also probed to enhance understanding. Each FGD audio-recording was transcribed verbatim, translated into English and entered into NVIVO, qualitative software for the purpose of analysis. A total of 80 men and 77 women inclusive of both intervention and control sites participated in the 16 FGDs conducted at baseline and 37 men and 38 women participated in the 8 FGDs conducted at follow-up.

### Intervention

**Figure 1:**
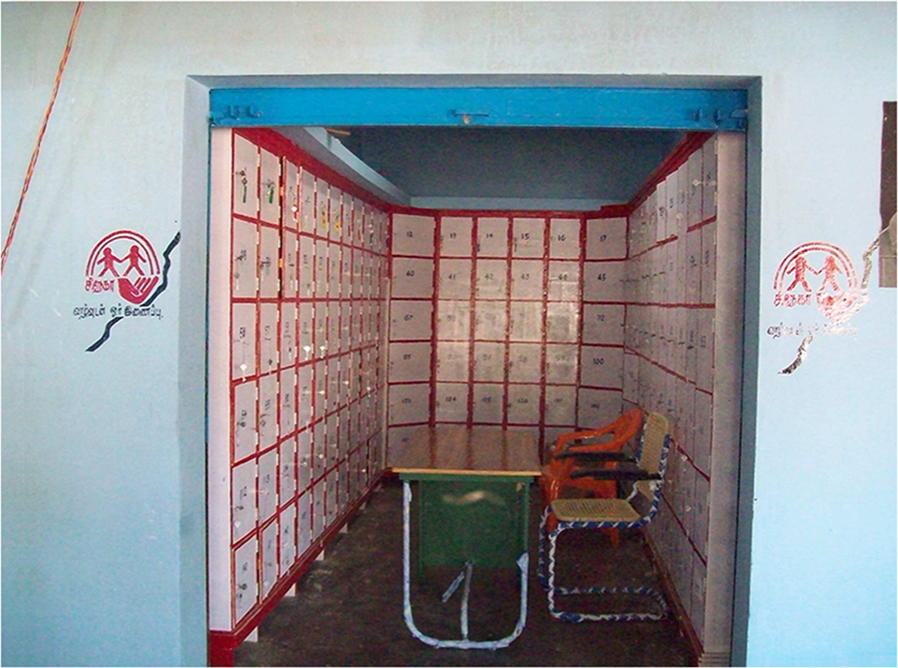
**Central storage facility.** The central storage facility with the boxes for pesticides storage.

Two centralized storage facilities (one in each village) were identified with the help of the Panchayat (local self-government). Within these buildings and based on available space 167 and 132 storage boxes (similar to a bank locker) were constructed. These boxes two feet by two feet in size, made of wood, were fixed to the wall and could not be removed from the facility. Each box could be locked (Figure 1). The cost of construction of the two central storage facilities was Rs. 95,000 (USD 1,500). The maintenance costs per month per facility was Rs.7,150 (USD 115). With the help of the Panchayat and other key persons in the villages, public meetings were organized to create awareness among residents about the storage facilities, their purpose and benefits. Farmers had access to their pesticide storage boxes at any time during the day from about 7 AM in the morning till about 7 PM in the evening. They had a key to their own locker and a duplicate key was kept with the manager of the central storage facility. Four managers (two for each facility), were identified by the local community and were in charge of managing the facility. They were provided training on the importance of safe storage and disposal of pesticides and were given an orientation into the purpose of the storage facility, their role in managing it in terms of regular attendance so as not to cause hardship to the farmers*.* They also maintained a register where they recorded frequency of usage.

### Sample size

For the study, pesticide suicides included attempted and completed pesticide suicides and ‘all suicides’, included attempted and completed pesticide suicide and suicides by other methods. Suicide attempts are usually 10–40 times more frequent than completed suicides [14]. This fact, coupled with data from previous epidemiological studies [15,16] in rural Tamil Nadu, helped us arrive at an estimate of nearly 10/1000. Factoring in a power of 80, alpha error at 0.05, one- sided test and expecting 50% reduction, sample size required was estimated at 3578 persons in each arm.

### Procedures

Six trained research assistants carried out a baseline door to door survey of all households in the study sites using a structured interview schedule. This survey was done before the construction of the storage facilities. In addition to documenting socio-demographic characteristics of the household, information on types of pesticides used, pesticide storage and disposal, knowledge about health risks of pesticides were also obtained. History of alcoholism and mental disorder in the family were noted. Information on attempted or completed suicides and/or accidental deaths occurring in the family over the previous one and a half years was also obtained. All assessments carried out in the intervention sites were carried out in the control sites. The survey was repeated at the end of one and a half years in both sites. Verbal autopsies were conducted for all deaths occurring during the intervention period. This information was cross-checked with the death certificate which is issued by the district taluk office and which confirms cause of death. In case of suicide, psychological autopsy was carried out. A surveillance system involving monthly visits to physicians, health workers, teachers, hospitals and police stations located in the study area was carried out to document reports of attempted or completed suicides occurring during the study period.

### Statistical analysis

The primary analyses were done on an Intention to Treat (ITT) basis. The outcome of the study was pesticide suicides and all suicides. Data on baseline suicides were obtained retrospectively (past 1.5 years) and rates were calculated accordingly. Data on suicides obtained during the follow-up included suicide data gathered through the survey and through the surveillance. Change in suicide rates from baseline to follow-up for intervention and control sites were calculated separately and then comparison between intervention and control sites for the above change and 95% confidence interval were calculated. Per protocol analysis for individuals with complete follow up was also done. Though the sample size was decided based on the one sided hypothesis, the test of significance was done based on the two sided test. Data were analyzed using SAS version 9.2. The trial no is ISRCTN04912407.

## Results

### Socio-demographic characteristics

At baseline, 4446 individuals (1097 households) in the intervention and 3307 individuals (782 households) in the control sites were surveyed. The number of households surveyed at baseline was less compared to the numbers given to us by the DRO. The main reason for this was because the DRO recorded the number of households based on the number of ration cards issued to a household. The ration card is a scheme developed by the Public Distribution System in Tamil Nadu, which entitles each household to substantial quantities of subsidized food grains. To avail of these benefits many families though living together in one household and having a common kitchen show themselves as nuclear families thereby obtaining more than one ration card. This resulted in an increased number of households as per the DRO. During the survey, however, we defined a household as members of a family living together and sharing a common kitchen.

At follow-up, there were 4308 individuals (1063 households) in the intervention and 2673 individuals (632 households) in the control sites. There was an overall 10% loss to follow up in the study and a higher proportion of loss (19%) in the control sites which was due to migration of communities and non-availability of respondents. Age, sex and marital status of the individuals were nearly similar across intervention and control sites (Table 1). More participants in the control sites were non-literate (20.8%) as compared to those in the intervention sites (16%). The proportion of farmers were however higher in the intervention sites (18.7%) as compared to the control sites (14.7%).

**Table 1:** Characteristics of study participants at baseline

**Variable**	**Intervention**		**Control**	
	**(N = 4446)**		**(N = 3307)**	
	**n**	**%**	**n**	**%**
**Age**				
< 14	904	20.3	679	20.5
14 – 19	501	11.3	377	11.4
20 – 29	851	19.1	675	20.4
30 – 39	719	16.2	524	15.9
40 – 49	592	13.3	426	12.9
50 – 59	413	9.3	308	9.3
60 +	466	10.5	318	9.6
**Sex**				
Male	2245	50.5	1625	49.1
Female	2201	49.5	1682	50.9
**Education**				
Non-Literate	709	16.0	688	20.8
Primary and Middle School	1653	37.2	1275	38.6
High, Secondary, Graduate and above	1824	41.0	1145	34.6
Not Applicable	260	5.9	199	6.0
**Occupation**				
Farmer (lease,Owner & Agricultural labours)	830	18.7	487	14.7
Skilled, Unskilled	568	12.8	625	18.9
Housewife	727	16.4	535	16.2
Unemployed	369	8.3	375	11.3
Others	1692	38.1	1086	32.8
Not Applicable	260	5.9	199	6.0
**Marital status**				
Single	1982	44.6	1487	45.0
Married	2121	47.7	1529	46.2
Divorced, Separated & Widowed	343	7.7	291	8.8

Households in the control sites (23.3%) reported higher levels of income as compared to those in the intervention sites (19.8%). A greater proportion of households in the intervention sites (75%) reported being in debt as compared to those in the control sites (55.8%). Households in the intervention sites reported possessing more land (49.6%) and using more pesticides (50.6%) as compared to those in the control sites (44.1% and 38.9% respectively) (Table 2).

**Table 2:** Characteristics of households at baseline

**Variable**	**Intervention**		**Control**	
	**(N = 1097)**		**(N = 782)**	
	**n**	**%**	**n**	**%**
**Income (INR)**				
< = 5000	174	15.9	29	3.7
5001 – 10,000	203	18.5	75	9.6
10,001 – 15,000	313	28.5	318	40.7
15,001 – 20,000	190	17.3	178	22.8
>20,000	217	19.8	182	23.3
**Does your family own land?**				
Yes	544	49.6	345	44.1
No	535	48.8	437	55.9
Land on lease	18	1.6	-	-
**In the last two years do you have any debts to be paid off?**				
Yes	823	75.0	436	55.8
No	274	25.0	346	44.3
**Do you use pesticides of any kind?**				
Yes	555	50.6	304	38.9
No	503	45.9	399	51.0
Don’t Know	39	3.6	79	10.1

### Acceptability of storage facility

A total of 248 households utilized the storage facility (23.3% of households). One hundred and thirty four respondents reported using the storage facility all or most of the time because they found it to be useful, safe and, conveniently located. Reasons for its usefulness were attributed to i) safe storage (62%) ii) time and cost saved in travel (60%) and (iii) other reasons like safety, theft, damage etc. (85%). The remaining 815 households out of a total of 1063 households at follow-up did not use the facility. Of this 447 (42%) did not own any land. Another 368 (35%) households did not use the facility because it was located too far from either their homes (more than 2 km) or their fields; and did not feel the need for a storage facility as they bought only as much as was needed or because they were not aware of it. The maximum utilization of the storage boxes was 94% in one village and 74% in the other and dropped to 43% and 29% during the monsoon. The storage of pesticides in the homes dropped from 44% at baseline to 7% at follow –up. In the control sites too storage of pesticides in homes dropped from 54% at baseline to 25% at follow- up.

The findings from the FGDs, revealed that participants were largely appreciative of the storage facility both as a means of preventing suicides and for providing a safe place for storing their pesticides. Most participants corroborated the survey findings when they spoke of the advantages of the central storage facility and said, “*Usually we used to buy only 2 spray cans worth of pesticides before, but now after this implementation of storage facility we are buying 6 spray cans of pesticides since we are now able to keep it safe in this centre”.***(Kandhamangalam, Men)**. They spoke of avoiding considerable wastage which usually occurred when left over pesticides were stored in the fields *“Pesticides being buried and wastage of it can now be saved…. then the quality of the pesticides is also retained. If it’s buried in the soil the effect of it is less, there is no such problem in storing at centre and there are no cases of forgetting the pesticides too”*. **(Kandhamangalam, Men)**. A few participants, however, expressed their reservations about the usefulness of the storage facility when they said, “*No one is responsible for any one; each one has their own thinking no matter what one says at the end of the day he/she is going to do what he/she thinks. If a person has decided that he or she wants to die they can never be saved. It can never be averted but maybe it can be reduced”***(Karunagaranallur women)**.

### Feasibility of the storage facility

Despite the above concerns, people’s attitude towards the storage facility was largely positive. The need for setting up more such facilities so as to benefit a larger number of farmers was expressed, *“There is a need for more CSFs in the villages to help people whose fields are further away”, (***Kandamangalam men).** Another participant suggested that the, *“Government should take over this* (the CSFs) *and make it compulsory for every farmer to store their pesticides here”, (***Kurungudi men).** But the most telling statement attesting to the value people attributed to the storage facility came from one farmer who said, “*I am alive today because of the CSF. Two months back, I had consumed some alcohol, went home and fought with my wife. I became really upset and wanted to consume pesticide and die. I was searching for it and after some time, my wife reminded me that I won’t find it at home as all our pesticides are kept in the CSF. I am alive today because of the centralized storage facility*” **(FGD, Male).** A woman participant went on to add that the presence of the storage facility was a great help to families not just because it had helped to “reduce suicides” but also because “....*, small children may consume it without their knowledge, now there is no chance for that. …..If the family has some problem especially between the husband and wife when they see the pesticide it will trigger them to drink the pesticide. Now there is no chance for that because they are keeping it in the storage room”****(*Kurungudi Women).**

### Pesticide suicides

Village specific attempted and completed pesticide suicide rates for intervention and controls sites are given in Table 3. With respect to attempted pesticide suicides at baseline there were 16 cases in the intervention sites and 5 in the control sites. At follow-up there were 3 in the intervention and 2 in the control sites. With respect to completed pesticide suicides, at baseline there were 10 cases in the intervention sites while there were none in the control sites. At follow up there were 2 cases in the intervention and 2 in the control sites. There were more deaths in the control site observed during the follow up period. None of the persons who had attempted or completed suicide in the intervention sites had utilized the central storage facility.

**Table 3:** No. of Pesticide Suicides (attempts and completed)

	**Baseline**			**Follow up**		
**Village name**	**No. of individuals**	**Pesticide suicides**	**Rate /100,000/year**	**No. of individuals**	**Pesticide suicides**	**Rate /100,000/year**
**Attempted suicide**						
**Intervention**						
Kandamangalam	2486	8	214.5	2376	1	28.1
Kurungudi	1960	8	272.1	1932	2	69.0
**Control:**						
Karunagaranallur	1398	1	47.7	1360	1	49.0
Pazhanjanallur	1909	4	139.7	1313	1	50.8
**Completed Suicide**						
**Intervention:**						
Kandamangalam	2486	9	241.3	2376	0	0
Kurungudi	1960	1	34.0	1932	2	69.0
**Control:**						
Karunagaranallur	1398	0	0.0	1360	1	49.0
Pazhanjanallur	1909	0	0.0	1313	1	50.8

The ITT analysis is presented in Table 4. With regard to attempted pesticide suicides, the rates of change from baseline to follow up in the intervention sites was nearly 292/100,000 individuals. In the control sites this was nearly 91/100,000 individuals. The difference in change from intervention to control site for one year was 135/100,000 individuals (95% CI: 8.5, 260.5) (p < 0.05). With regard to completed pesticide suicides, the rates of change from baseline to follow up in the intervention and control sites were nearly 180/100,000 and −60/100,000 individuals respectively. The reduction in completed pesticide suicides per year was 160/100,000 individuals (95% CI: 98.9, 221.7) (p < 0.001).

**Table 4:** Change in pesticides suicide after intervention *

	**Intervention**			**Control**			**Difference in change**	**95% CI**	
	**Total population**	**n**	**Rate /100,000**	**Total population**	**n**	**Rate /100,000**			**P-value**
**Attempted suicide:**									
Baseline	4446	16	359.9	3307	5	151.2			
Follow up	4308	3	69.6	2673	2	74.8			
Change 1½ yr		13	292.4		3	90.7	201.7	(12.7,390.7)	<0.05
Change 1 yr							134.5	(8.5, 260.5)	<0.05
**Completed suicide:**									
Baseline	4446	10	224.9	3307	0	0			
Follow up	4308	2	46.4	2673	2	74.8			
Change 1 ½ yr		8	179.9		−2	−60.5	240.4	(148.3, 332.6)	<0.001
Change 1 yr							160.3	(98.9, 221.7)	<0.001
**All pesticide suicides:**									
Baseline	4446	26	584.8	3307	5	151.2			
Follow up	4308	5	116.1	2673	4	149.6			
Change 1 ½ yr		21	472.3		1	30.2	442.1	(232.0 , 652.2)	<0.001
Change 1 yr							294.7	(154.7, 434.8)	<0.001

*Note:

1. Pesticide Suicides included attempted and completed pesticide suicides.

2. Change calculated based on population at baseline.

We also analyzed pesticide suicides (attempted and completed). There were 26 cases in the intervention sites at baseline and 5 in the control sites. At follow up there were 5 in the intervention sites and 4 in the control sites. The rates of change from baseline to follow up in the intervention and control sites were 472/100,000 and 30/100,000 respectively. The difference in change when calculated for a year was 295/100,000 individuals (95% CI: 154.7, 434.8) (p < .001).

### All suicides

There were 33 cases of all suicides in the intervention sites at baseline and 10 in the control sites (Table 5). At follow up there were 5 cases of all suicides in the intervention sites and 6 in the control sites. The rates of change from baseline to follow up in the intervention and control sites were 630/100,000 and 121/100,000 respectively. The difference in change when calculated for a year was 339/100,000 individuals (95% CI: 165.3, 513.2, p < .001).

**Table 5:** Change in all suicides after intervention*

	**Intervention**			**Control**			**Difference in change**	**95% CI**	
	**Total population**	**n**	**Rate /100,000**	**Total population**	**n**	**Rate /100,000**			**P-value**
**Attempted suicide:**									
Baseline	4446	20	449.8	3307	7	211.7			
Follow up	4308	3	69.6	2673	4	149.6			
Change 1½ yr		17	382.4		3	90.7	291.7	(83.2, 500.1)	<0.05
Change 1 yr							194.5	(55.5, 333.4)	<0.05
**Completed suicide:**									
Baseline	4446	13	292.4	3307	3	90.7			
Follow up	4308	2	46.4	2673	2	74.8			
Change 1½ yr		11	247.4		1	30.2	217.2	(59.6, 374.8)	<0.05
Change 1 yr							144.8	(39.7, 249.9)	<0.05
**All suicides:**									
Baseline	4446	33	742.2	3307	10	302.4			
Follow up	4308	5	116.1	2673	6	224.5			
Change 1½ yr		28	629.8		4	121.0	508.8	(247.9, 769.8)	<0.001
Change 1 yr							339.2	(165.3, 513.2)	<0.001

*Note:

1. Suicides include attempted and completed pesticide suicide and suicides by other methods.

2. Change calculated based on population at baseline.

The rates of change were also calculated based on per protocol analysis. For pesticide suicides the difference in change was 300/100,000 individuals (95% CI: 153.0, 447.1, p < .01) while for all suicides it was 334/100,000 individuals (95% CI: 146.1, 521.0, p < .01) similar to the rates emerging from the ITT analyses.

### Verbal and psychological autopsy

There were totally 12 deaths (6 males and 6 females) in the intervention and 8 deaths (5 males and 3 females) in the control sites. There were two suicides each in the intervention and control sites. Both suicides in the intervention sites were by women, one of whom had an Axis I diagnosis of depression, with a situational stress of physical abuse by an alcoholic spouse The other woman had depressive symptoms for duration of six days only as she was being forced into marrying a widower by her family. In the control site, both suicides were by men, one of whom had an Axis I diagnosis of alcohol dependence. The second man committed suicide after his girlfriend left him.

## Discussion

This study, the first of its kind in the world, attempted to examine the feasibility and acceptability of a central storage facility as a possible means of limiting access to pesticides thereby reducing pesticide suicides. Households who used the facility found it to be useful, safe and, conveniently located. Following the intervention, the differences between sites in changes in combined, completed and attempted suicide rates per 100,000 person-years were 295 (95% CI: 154.7, 434.8; p < 0.001) for pesticide suicide and 339 (95% CI: 165.3, 513.2, p < 0.001) for suicide of all methods.

### Pesticide suicides

In India the National Crimes Record Bureau [13] (NCRB), reported that 21,084 suicides were by pesticide ingestion. According to Patel et al. [12] deaths caused by pesticide poisoning amounted to 92,000 with about 90% of all suicides occurring in rural areas and 10.3% involving farmers and agricultural workers. This suggests that the government data is a gross under estimation. Given that agriculture constitutes a major component of India’s economy and comprises 70% of the country’s workforce, pesticides are used widely. This widespread use of pesticides in rural areas implies its easy accessibility and an easy means for suicide. Over 80% of cases of deliberate self harm and suicides by ingestion of pesticides were reported in studies from, rural Tamil Nadu and West Bengal, [15,17]. The study from China [4] found that 65% of pesticide suicides used chemicals stored in the home, thus enhancing access. Mohammed et al. [18] in Sri Lanka found that over 76% of patients admitted with intentional pesticide ingestion had stored the pesticides either inside or immediately outside the house. Our baseline study findings revealed that a substantial proportion of respondents in the intervention and control sites had stored their pesticides in fields and within their homes and these were not usually kept locked.

Limiting access to lethal means and methods of self-harm, referred to as “means restriction”, has increasingly been found to be a useful suicide prevention strategy [4,19,20]. Murray and Taylor [21] suggested installing locked storage cabinets for pesticides as a means of reducing pesticide poisoning. Commenting on the individual boxes for storage of pesticides that they had provided in their study, Hawton et al. [9] reported fairly consistent and responsible use of these boxes by households. Pearson et al. [22] have reported on a large effectiveness study of individual storage boxes underway in rural Sri Lanka wherein 44,000 households will be participating in a randomized controlled trial and followed up over a three year period. The findings emerging from this study may provide useful insights into the effectiveness of these boxes in preventing pesticide suicides. In another study, an evaluation of four villages in Andhra Pradesh, India, that had stopped using chemical pesticides in favour of non-pesticide management (NPM), found that restriction of pesticide availability and use of NPM had the potential to reduce pesticide suicides [23].

According to Konradsen [5] a unique feature common to developing countries, particularly those in Asia is the use of pesticides for self-harm. Gunnell [8] recommends that the most practical suicide prevention strategy would be to reduce access to organophosphate pesticides which combined with public education would prove an effective pesticide suicide prevention strategy. A study from Taiwan has found that a reduction in pesticide suicide has not resulted in a concomitant increase in suicide rates by other methods (methods substitution) [24]. Chang et al. [25] while reporting on pesticide suicides in Taiwan said that higher pesticide suicide rates were evident in places where a larger proportion of people worked in agriculture. The authors surmised that easy access to pesticides contributed to higher pesticide suicide rates and underscored the need for targeted prevention strategies like restricting access to pesticides as a means of protecting against pesticide suicides.

Typically the most common reasons for suicides were financial crisis and debts, unhappy family relations and problems in the family, alcoholic husbands and failure in love [26-28]. Domestic violence and sexual abuse have also been reported as common causative factors for suicides the world over [29,30]. Results from the psychological autopsies from our study also highlighted the role of alcohol abuse and domestic violence, as important factors for suicide.

### Feasibility and acceptability

The storage facility may have had a role in minimizing suicides in the intervention sites. The reduction was 160 completed suicides and 135 attempted suicides by pesticide ingestion per 100,000 populations per year. Important to note is the fact that in all those cases of attempted (n = 3) and completed (n = 2) suicides that took place in the intervention sites at follow-up, the pesticides had not been stored in the storage facility. The study from Sri Lanka, demonstrated that people appreciated the storage boxes but because these were kept at home and not in the fields it was likely to increase the risk of impulsive self-poisoning episodes owing to its easy accessibility. Further, the boxes were not always kept locked [10]. Our study involved constructing a community storage facility wherein all farmers could store their pesticides and where they could ensure that their pesticides were safe. The results showed that the storage of pesticides in the homes dropped from 44% at baseline to 7% at follow -up, indicating greater awareness among people about the risks of storing pesticides at home.

The other important aspect of our intervention was the manner in which we went about setting it up. For any community based intervention to work efficiently, the need for community acceptance of the programme is critical. By seeking the involvement of the local panchayat leaders and other decision makers and engaging members of the community in discussions- as was done during the FGDs - we made the whole process more participatory. These discussion groups and subsequent meetings played an important role both in enhancing acceptance of the intervention and in infusing in the farmers a sense of ownership. Thus, it was they who decided the location of the facility, the supervision of its construction and finally the selection of two local persons charged with the responsibility of managing the facility. In the process the facility received adequate publicity among the households in the villages to the extent that people from neighbouring villages came over and asked us when we would be setting up a similar facility in their village. However, out of 562 farming households, only 248 households (23%) utilised the facility. Thirty five percent did not use the facility as they lived far away from the location. This suggests the need for making the central storage facility more accessible and convenient for farmers.

The data we gathered on suicides through surveillance contributed an additional 3 more cases of suicidal attempts and 2 cases of suicidal deaths, implying that people had been reluctant to provide information on suicides during the survey. None of the suicidal attempts or deaths had been reflected in the police and official records. Attempting suicide is a punishable offence under the Indian Penal Code and families fear disclosing information for fear of social stigma and harassment. The importance of de-criminalising suicides and building awareness among health care providers and the public about the risk of suicides, the need for intervention and, notification strategies are essential to reduce suicides.

A major limitation in our study was that the intervention was only carried out in two villages. A larger number of villages followed up over a longer time period would be necessary to prove effectiveness. Secondly, the baseline differences in the incidence of suicides between the intervention and control sites preclude our ability to establish the effectiveness of the storage facility as a pesticide suicide prevention strategy. Thirdly, the follow–up duration of one and a half years may not have been adequate to assess the sustainability of the storage facility. Fourthly, almost 19% loss to follow up in the control villages was another limitation which occurred due to migration of an entire community who had been residing in those villages. Despite these limitations, our study has provided preliminary findings on the feasibility and acceptability of the storage facility as a probable pesticide suicide prevention strategy.

## Conclusion

Suicide is a multifaceted problem and hence suicide prevention programmes should also be multidimensional. In India suicide prevention is viewed more as a social objective than a traditional exercise in the health sector. Reducing alcohol availability and consumption, unemployment, poverty, domestic violence, social inequities, increasing mental health awareness and improving mental health services are essential to reduce suicides. While a multi pronged approach addressing the above factors would be a necessary long term strategy, the central storage facility as described in this study, as a medium term strategy, is likely to be a feasible step for reducing pesticides suicides in developing countries. Further, the facility is simple, culturally acceptable and locally participative, thereby contributing to its sustainability. Future studies involving larger populations are necessary to assess the effectiveness of such storage facilities in a variety of settings.

## Competing interests

The authors declare that they have no competing interests.

## Authors’ contributions

LV conceived the study. LV, SK, RM and LJ contributed to the study design and conduct of study. MS did the data collection and data monitoring. LJ and DS were instrumental in data analysis and interpretation of data, and manuscript preparation. All authors participated in the drafting of the final article. All authors read and approved the final manuscript.

## Pre-publication history

The pre-publication history for this paper can be accessed here:

http://www.biomedcentral.com/1471-2458/13/850/prepub

## References

[B1] Bertolote JM Fleischhmann A Eddelston M Gunnell D Death from pesticides poisoning: a global response Br J Psychiatry 2006 189 201 203 10.1192/bjp.bp.105.020834 16946353 PMC2493385

[B2] Gunnell D Eddleston M Phillips MR Konradsen F The global distribution of fatal pesticide self-poisoning: systematic review BMC Public Health 2007 7 357 10.1186/1471-2458-7-357 18154668 PMC2262093

[B3] Gross VA Weiss MG Ring M Hepp U Bopp M Gutzwiller F Rössler W Methods of suicide: international suicide patterns derived from the WHO mortality database Bull World Health Organ 2008 86 657 736 10.2471/BLT.07.043489 PMC2649482 18797649

[B4] Phillips MR Yang G Zhang Y Wang L Ji H Zhou M Risk factors for suicide in China: a national case–control psychological autopsy study Lancet 2002 360 1728 1736 10.1016/S0140-6736(02)11681-3 12480425

[B5] Konradsen F Van der Hoek W Peiris P Reaching for the bottle of pesticide—a cry for help. Self-inflicted poisonings in Sri Lanka Soc Sci Med 2006 62 1710 1719 10.1016/j.socscimed.2005.08.020 16165259

[B6] Mishara BL Prevention of deaths from intentional pesticide poisoning Crisis 2007 28 suppl 1 10 20 10.1027/0227-5910.28.S1.10 26212190

[B7] Eddleston M Karalliedde L Buckley N Fernando R Hutchinson G Isbister G Konradsen F Murray D Piola JC Senanayake N Sheriff R Singh S Siwach SB Smit L Pesticide poisoning in the developing world-a minimum pesticides list Lancet 2002 360 1163 1167 10.1016/S0140-6736(02)11204-9 12387969

[B8] Gunnell D Fernando R Hewagama M Priyangika WD Konradsen F Eddleston M The impact of pesticide regulations on suicide in Sri Lanka Int J Epidemiol 2007 36 1235 1242 10.1093/ije/dym164 17726039 PMC3154644

[B9] Hawton K Ratnayeke L Simkin S Harriss L Scott V Evaluation of acceptability and use of lockable storage devices for pesticides in Sri Lanka that might assist prevention of self-poisoning BMC Public Health 2009 9 69 10.1186/1471-2458-9-69 19239714 PMC2656540

[B10] Konradsen F Pieris R Weerasinghe M Van der Hoek W Eddleston M Dawson AH Community uptake of safe storage boxes to reduce self-poisoning from pesticides in rural Sri Lanka BMC Public Health 2007 26 7 13 10.1186/1471-2458-7-13 PMC1796869 17257415

[B11] Konradsen F Dawson AH Eddleston E Gunnell D Pesticide self-poisoning: thinking outside the box Lancet 2007 369 9557 169 170 10.1016/S0140-6736(07)60085-3 17240268 PMC1963473

[B12] Patel V Ramasundarahettige C Vijayakumar L Thakur JS Gajalakshmi V Gururaj G Suraweera W Jha P Suicide mortality in India: a nationally representative survey Lancet 2012 379 2343 2351 10.1016/S0140-6736(12)60606-0 22726517 PMC4247159

[B13] National Crimes Records Bureau Suicides in India. In: Accidental Death and Suicides in India. Ministry of Home Affairs 2011 Downloaded from: http://ncrb.nic.in/CD-ADSI2009/suicides-09.pdf. Accessed on 3rd July 2012

[B14] Schmidtke A Bille-Brahe U DeLeo D Kerkhof A Bjerke T Crepet P Haring C Hawton K Lönnqvist J Michel K Pommereau X Querejeta I Phillipe I Salander-Renberg E Temesváry B Wasserman D Fricke S Weinacker B Sampaio-Faria JG Attempted suicide in Europe: rates, trends and socio-demographic characteristics of suicide attempters 1989–1992. Results of the WHO/EURO Multi centre study on parasuicide Acta Psyciatr Scand 1996 93 327 338 10.1111/j.1600-0447.1996.tb10656.x 8792901

[B15] Gajalakshmi V Peto R Suicide rates in rural Tamil Nadu, South India. Verbal autopsy of 39,000 deaths in 1997–98 Int J Epidemiol 2007 36 203 207 10.1093/ije/dyl308 17301103

[B16] Aaron R Joseph A Abraham S Muliyil J George K Prasad J Minz S Suicides In young people in rural southern India Lancet 2004 363 1117 1118 10.1016/S0140-6736(04)15896-0 15064031

[B17] Banerjee S Chowdhury AN Scheling L Brahma A Biswas MK Weiss MG Deliberate self harm and suicide by pesticide ingestion in the Sundarban region, India Trop Med Int Health 2009 14 213 219 10.1111/j.1365-3156.2008.02199.x 19236667

[B18] Mohamed F Manuweera G Gunnel D Azher S Eddleston M Dawson A Konradsen F Pattern of pesticide storage before pesticide self poisoning in rural Sri Lanka BMC Public Health 2009 9 405 9 405 10.1186/147/2458 19889236 PMC2777873

[B19] Graafsma T Kerkhof A Gibson D Badloe R van de Beek LM High rates of suicide and attempted suicide using pesticides in Nickerie, Suriname South America Crisis 2006 27 77 81 16913329 10.1027/0227-5910.27.2.77

[B20] Konradsen F van der Hoek W Cole DC Hutchinson G Daisley H Singh S Eddleston M Reducing acute poisoning in developing countries – options for restricting the availability of pesticides Toxicol 2003 192 249 261 10.1016/S0300-483X(03)00339-1 14580791

[B21] Murray DL Taylor PL Claim no easy victories: evaluating the pesticide industry’s Global Safe Use campaign World Dev 2000 28 1735 1749 10.1016/S0305-750X(00)00059-0

[B22] Pearson M Konradsen F Gunnell D Dawson AH Pieris R Weerasinghe M Knipe DW Jayamanne S Metcalfe C Hawton K Wickramasinghe AR Atapattu W Bandara P De Silva D Ranasinghe A Mohammed F Buckley NA Gawarammana J Eddleston M A community-based cluster randomised trial of safe storage to reduce pesticide self-poisoning in rural Sri Lanka: study protocol BMC Public Health 2011 11 879 10.1186/1471-2458-11-879 22104027 PMC3227631

[B23] Vijayakumar L Satheesh–Babu R Does ‘No Pesticide’ Reduce Suicides? Int J Soc Psych 2008 55 5 401 406 10.1177/0020764008095340 19617276

[B24] Lin J-J Lu T-H Trends in solids/liquids poisoning suicide rates in Taiwan: test of the substitution hypothesis BMC Public Health 2011 11 712 10.1186/1471-2458-11-712 21933432 PMC3182937

[B25] Chang S-S Tsung-Hsueh L Sterne JAC Eddleston M Lin J-J Gunnell D The impact of pesticide suicide on the geographic distribution of suicide in Taiwan: a spatial analysis BMC Public Health 2012 12 260 10.1186/1471-2458-12-260 22471759 PMC3351735

[B26] Vijayakumar L Rajkumar S Are risk factors for suicide universal? A case–control study in India Acta Psychatr Scand 1999 99 6 407 411 10.1111/j.1600-0447.1999.tb00985.x 10408261

[B27] Kumar S Jeyaseelan J Suresh S Ahuja RC Domestic violence and its mental health correlates in Indian women Br J Psychiatry 2005 187 62 77 10.1192/bjp.187.1.62 15994573

[B28] Manoranjitham SD Rajkumar AP Thangadurai P Prasad J Jayakaran R Jacob KS Risk factors for suicide in rural south India Br J Psychiatry 2010 196 26 30 10.1192/bjp.bp.108.063347 20044655

[B29] Heise L Garcia-Moreno C Krug EG, Dahlberg LL, Mercy JA Violence by intimate partners World Report on Violence and Health 2002 Geneva: WHO 89 121

[B30] Maris RW Suicide Lancet 2002 360 319 26 10.1016/S0140-6736(02)09556-9 12147388

